# Polysaccharide synthesis operon modulates *Rickettsia*-endothelial cell interactions

**DOI:** 10.1371/journal.ppat.1013277

**Published:** 2025-06-26

**Authors:** Smruti Mishra, Luke Helminiak, Hwan Keun Kim

**Affiliations:** 1 Center for Infectious Diseases, Stony Brook University, Stony Brook, New York, United States of America; 2 Department of Microbiology and Immunology, Stony Brook University, Stony Brook, New York, United States of America; Virginia Commonwealth University School of Medicine, UNITED STATES OF AMERICA

## Abstract

Pathogenic *Rickettsia* species target vascular endothelial cells and cause systemic vasculitis. As obligate intracellular bacterial pathogens, *Rickettsia* must secure nutritional resources within the cytoplasm of endothelial cells while simultaneously subverting the innate immune defense system. With advances in rickettsial and host genetics, recent studies have identified novel molecular mechanisms involved in the complex interactions between *Rickettsia* and endothelial cells. However, it remains unclear how *Rickettsia* shields pathogen-derived immune stimulants, such as lipopolysaccharides (LPS) and peptidoglycan fragments, from immune recognition during intracellular replication. Prior work described two *Rickettsia conorii* variants with *kkaebi* transposon insertions in the *polysaccharide synthesis operon* (*pso*). Biochemical and immunological analyses revealed that *pso* is responsible for the biosynthesis of O-antigen (O-Ag) and the proper assembly of surface proteins. In the present work, we document that *pso* variant HK2 exhibits reduced capacities to adhere to and invade microvascular endothelial cells. Despite the low intracellular abundance, HK2 induced significantly higher levels of proinflammatory cytokines and chemokines, leading to premature cell death. Notably, HK2 exhibited defective intracellular survival in bone marrow-derived macrophages. This inability to dampen endothelial cell-mediated immune stimulation and resist macrophage-induced bactericidal activities resulted in the rapid elimination of viable *Rickettsia* in the mouse model of spotted fever. Further, when tested as a live-attenuated vaccine, HK2 elicited robust protective immunity against lethal spotted fever pathogenesis. Our work highlights the crucial role of *pso* in enabling *Rickettsia* to evade immune surveillance during intracellular replication within endothelial cells, ultimately delaying pathogen-induced programmed cell death and escaping immune defense mechanisms.

## Author summary

*Rickettsia* exhibits an obligate intracellular lifecycle, circulating between mammalian hosts and arthropod vectors. In human patients, pathogenic *Rickettsia* species target vascular endothelial cells and cause systemic vasculitis, leading to lethal outcomes without prompt antibiotic treatment. Strikingly, despite the ongoing reductive genome evolution, pathogenic *Rickettsia* species have evolved to evade endothelial immune detection mechanisms and exploit macrophage immune responses for their survival. Unfortunately, the factors enabling rickettsial intracellular survival are incompletely understood. Thus, additional insights into the underlying molecular mechanisms will assist in developing vaccines and therapeutics for rickettsioses. We carried out a series of tissue culture and animal infection studies in which we compared the virulence of *Rickettsia conorii* transposon insertional mutants expressing antigenically distinct O-Ag, a critical constituent of LPS. Our experimental results suggest that rickettsial O-Ag plays an important role during the intracellular lifecycle in endothelial cells and macrophages, contributing to immune evasion and systemic vasculitis. Further, we report that, without O-Ag, *R. conorii* exhibited significantly reduced virulence with excellent potency to elicit protective immunity against spotted fever pathogenesis. Our studies provide a strong foundation for immunological studies defining rickettsial innate immune evasion strategies and host immunity against rickettsioses.

## Introduction

*Rickettsia* species are Gram-negative, obligate intracellular α-proteobacteria that circulate between hematophagous arthropod vectors (e.g., ticks, fleas, mites, and lice) and vertebrate hosts. Recent genomic studies categorized the genus *Rickettsia* into four groups: the spotted fever group (SFG), typhus group (TG), transitional group, and ancestral group [1]. Clinical rickettsioses typically begin with a sudden onset of fever with a wide range of “flu-like” non-specific symptoms [2]. Clinical findings, bioinformatic analyses, and laboratory studies indicate that rickettsial species display varying pathogenicity and clinical manifestations [3,4]. Despite the differences in genome architecture and virulence, vasculitis forms the basis of rickettsial pathogenesis [5–7]. After vector transmission, pathogenic *Rickettsia* species preferentially target vascular endothelial cells for intracellular replication and immune evasion. Infections with highly pathogenic rickettsial species, such as *Rickettsia rickettsii* (Rocky Mountain spotted fever) and *Rickettsia conorii* (Mediterranean spotted fever), rapidly progress to systemic and lethal vasculitis without prompt antibiotic (doxycycline) intervention [8,9].

Endothelial cells play critical biological roles in the cardiovascular and lymphatic circulatory systems, acting as a tightly regulated semipermeable barrier to the underlying tissues. For instance, endothelial cells govern vascular permeability, maintain tissue homeostasis, modulate local and systemic inflammatory responses, and contribute to host immunity [10]. By directly engaging with bloodborne pathogens and circulating toxins, endothelial cells activate innate immune signaling mechanisms, recruit professional immune cells to infection sites, and coordinate inflammatory responses [11]. With the establishment of *in vitro* endothelial infection models, studies have begun to understand the functional roles of endothelial cells in modulating rickettsial pathogenesis [12,13]. However, despite recent advancements in developing genetic tools for *Rickettsia*, there is a significant gap in understanding the rickettsial virulence factors involved in immune evasion and modulation of endothelial cells [14,15]. Understanding these interactions could reveal novel molecular pathways involved in rickettsioses and aid in developing targeted therapeutic strategies.

Lipopolysaccharide (LPS) is an immunomodulatory virulence factor that supports the integrity of the outer membrane in Gram-negative bacteria [16,17]. LPS can be divided into three functional units: the conserved lipid A, which has varying numbers and lengths of acyl chains (endotoxin), the core oligosaccharide, and the O-antigen (O-Ag), which consists of varying lengths of chemically distinct repeating units. Mammalian hosts use multiple and comprehensive strategies to detect LPS and shape appropriate immune responses against invading pathogens, creating selective pressure that drives the development of immune-evasive strategies [18,19]. Consequently, Gram-negative bacteria have evolved to produce diverse LPS molecules with altered chemical and immunological properties and synthesize effector molecules to counteract LPS-driven immune signaling pathways in host cells [20].

In the order Rickettsiales, only members of the genus *Rickettsia* synthesize a complete Gram-negative cell envelope that contains both LPS and peptidoglycan, utilizing host-derived metabolites [21,22]. Rickettsial lipid A molecules are structurally distinct from those of other Gram-negative bacteria and possess longer acyl chains compared to the highly inflammatory lipid A found in *Escherichia coli* [23]. Moreover, structural diversity has been observed in recently diverged SFG rickettsiae, such as *R. rickettsii*, which generates lipid A with short 2’ secondary acyl chains [23,24]. Since lipid A influences the integrity of the bacterial outer membrane and regulates endotoxic capacity, changes in lipid A structure may significantly affect rickettsial virulence. On the other hand, O-Ag synthesized by SFG and TG rickettsiae are highly antigenic, elicit distinct serologic responses, tether surface proteins, and contribute to pathogenesis [25–27]. Recent studies have highlighted complex host-pathogen interactions through which multiple LPS-induced immune pathways contribute to rickettsial immune modulation and pathogenesis [28–30]. However, despite the significant importance of LPS in bacterial pathogenesis, how *Rickettsia* utilizes LPS for its pathogenesis and vector transmission remains largely unknown.

Our previous work established that the polysaccharide synthesis operon (*pso*) is conserved in *Rickettsia* and contributes to the biosynthesis of O-Ag, surface protein assembly, host cell attachment, and spotted fever pathogenesis [25]. However, the molecular functions of *pso* during intracellular replication in endothelial cells and macrophages have remained undetermined. In this study, we report that *pso* plays a pivotal role in intracellular survival and dampening endothelial cell responses to rickettsial infections. Without *pso*, rickettsial infections induced robust inflammatory responses in endothelial cells, leading to premature cell death. Additionally, unlike wild-type (WT) infections, macrophages restricted the intracellular replication of the *pso* variant HK2. Corroborating these results, mouse infection studies determined that HK2 is avirulent and, when used as a live-attenuated vaccine, elicits protective immunity in mice.

## Materials and methods

### Ethics statement

This study was carried out in strict accordance with the recommendations in the Guide for the Care and Use of Laboratory Animals of the NIH. Research was performed in accordance with institutional guidelines following experimental protocol review, approval, and supervision by the Institutional Biosafety Committee (IBC, 1445506) and Institutional Animal Care and Use Committee (IACUC, 1456687) at Stony Brook University. The Institutional Biosafety Committee and the National Institute of Health Recombinant DNA Advisory Committee reviewed and approved the use of a gene encoding chloramphenicol resistance for selecting mutants with insertional *kkaebi* lesions in *R. conorii*. The Division of Animal Laboratory Research at Stony Brook University operates in accordance with the American Association for Laboratory Animal Science, the American College of Laboratory Animal Medicine, and Animal Welfare Assurance ID D16-00006 (A3011-01) of the NIH. Experiments with infectious *Rickettsia* were performed in animal biosafety level 3 containment. Mice were housed in either standard filter-topped shoebox micro-isolator cages or filter-ventilated cages. Rooms had 10–15 air changes per hour and were maintained at 70–72°F. Pelleted irradiated Purina mouse chow was provided *ad libitum*, and hyper-filtered water (2 μm) was provided via water bottles *ad libitum*. All mice were provided with Enviro-Dri nesting material. Mice were euthanized with 3 liter·min^-1^ carbon dioxide inhalation, consistent with the recommendations of the panel on euthanasia of the American Veterinary Medical Association and Stony Brook University IACUC.

### Tissue culture cells and *Rickettsia*

Vero cells (African green monkey kidney epithelial cells, ATCC) were cultured in Dulbecco’s modified Eagle’s medium (DMEM, Sigma) supplemented with 10% heat-inactivated fetal bovine serum (HI-FBS, Gibco) at 37°C in a 5% CO_2_ atmosphere. Human microvascular endothelial cells (HMEC-1, ATCC) were cultured in Molecular, Cellular, and Developmental Biology medium (MCDB 131, Sigma) supplemented with epidermal growth factor (10 ng·ml^-1^, R&D Systems), hydrocortisone (1 µg·ml^-1^, Sigma), and 10% HI-FBS. Human dermal microvascular endothelial cells (HDMEC, PromoCell) were grown in endothelial cell basal medium (PromoCell) mixed with supplement pack (PromoCell) at 37°C in a 5% CO_2_ atmosphere. Stocks of *R. conorii* strain Malish 7 (ATCC) were generated by growing them in Vero cells at 34°C in a 5% CO_2_ atmosphere and subjecting to differential centrifugation through 25% MD-76R solution (816 mM meglumine diatrizoate, 157 mM sodium diatrizoate hydrate, 1 mM NaH_2_PO_4_, pH 7.0; 21,000 × g, 4°C, 45 min). Rickettsial samples were stored at -80°C in SPG buffer (218 mM sucrose, 3.8 mM KH_2_PO_4_, 7.2 mM K_2_HPO_4_, 4.9 mM L-glutamate, pH 7.2). *pso* variants were grown in Vero cells with chloramphenicol (0.3 µg·ml^-1^). Rickettsial genomic DNA samples were Illumina sequenced on a NextSeq 550 instrument to confirm their sequence identities (PureLink Genomic DNA Mini Kit, Invitrogen, NCBI Reference Sequences: NC_003103.1).

### Bone marrow-derived macrophages (BMDMs)

BMDMs were isolated from the femurs and tibias of C3H/HeN mice (male and female, Charles River Laboratories). Femurs and tibias were flushed with Dulbecco′s phosphate-buffered saline (DPBS, Sigma) supplemented with penicillin (100 units·ml^-1^, Gibco) and streptomycin (100 µg·ml^-1^, Gibco). After brief centrifugation (150 × *g*, 4°C, 5 minutes), cell pellets were resuspended in DMEM supplemented with 1 mM sodium pyruvate (Gibco), 20% HI-FBS, and 30% L929-conditioned media [filter-sterilized media prepared from L929 cells (ATCC) grown in minimal essential medium supplemented with 2 mM L-glutamine (Corning), 1 mM sodium pyruvate, 1 mM non-essential amino acids (Gibco), and 10% HI-FBS, at 37°C in a 5% CO_2_ atmosphere for 10 days]. The resuspended cells were seeded into culture dishes and incubated at 37°C in a 5% CO_2_ atmosphere for 5 days. Next, the cells were detached using ice-cold DPBS, resuspended in the culture medium, and plated onto 6-well plates (5 × 10^5^ cells per well). The attached cells were infected with *Rickettsia* in DMEM supplemented with 1 mM sodium pyruvate, 10% HI-FBS, and 15% L929-conditioned media at 34°C in a 5% CO_2_ atmosphere.

### Tissue culture infections with *Rickettsia*

Growth curves were generated by infecting monolayers of Vero, HMEC-1, HDMEC, or BMDM with *R. conorii* WT or *pso* variants in 6-well plates at a multiplicity of infection (MOI) of 0.01 (Vero, HMEC-1, or HDMEC) or 0.1 (BMDM). At 2-day (Vero, HMEC-1, or HDMEC) or 1-day (BMDM) intervals, cells in each well were dislodged with sterile glass beads and lysed by vortexing with glass beads. Infectious titers were determined by infecting monolayers of Vero cells with 10-fold serial dilutions of cell lysates or mouse organ homogenates containing *Rickettsia* in DMEM supplemented with 5% HI-FBS. Upon infection, Vero cells were incubated for 1 hour at 34°C with 5% CO_2_ to allow attachment and overlaid with DMEM containing 5% HI-FBS and 0.5% agarose. For organ homogenate samples, the growth media were supplemented with ampicillin (25 µg·ml^-1^, lung, Sigma) and/or amphotericin B (0.5 µg·ml^-1^, lung and spleen, Corning). Plaque numbers were determined by day 7 post-infection (pi).

### Microscopy analysis

Cytotoxicity was assessed for Vero, HMEC-1, or HDMEC cells infected with *Rickettsia* using 4 µM ethidium homodimer-1 in PBS (Invitrogen, Vero and HMEC-1) or by quantifying the areas exhibiting cytopathology (HDMEC, normalized by the areas formed by *R. conorii* WT). After incubating the plates for 30 minutes at room temperature, fluorescent and differential interference contrast images were acquired using NIS-Elements Basic Research software (Nikon). All images were contrast-adjusted and analyzed using ImageJ (NIH).

### Lactate dehydrogenase (LDH) assay

HMEC-1 cells were infected with *R. conorii* or *pso* variants and incubated at 34°C with 5% CO_2_. At predetermined time points, LDH abundance in the supernatant of mock- or *Rickettsia*-infected HMEC-1 cells was measured (CytoTox 96 Non-Radioactive Cytotoxicity Assay, Promega). Percent LDH release was calculated by subtracting the absorbance of the media-only control and normalizing it to the maximum LDH release (uninfected HMEC-1 cells treated with lysis buffer).

### Microarray analyses

HMEC-1 cells were infected with *R. conorii* or *pso* variants at an MOI of 3 for 6 hours at 34°C with 5% CO_2_. After washing the HMEC-1 cells, total RNA was isolated (miRNeasy Mini kit, Qiagen) to prepare samples for whole transcriptome expression analysis (150 ng, GeneChip WT PLUS Reagent Kit, Thermo Fisher Scientific). A hybridization cocktail containing 1.6 μg of fragmented and labeled single-stranded cDNA was hybridized to Clariom S Human Arrays (Thermo Fisher Scientific) for 16 hours at 45°C (60 rpm, GeneChip Hybridization Oven 640). Once the hybridization was complete, the arrays were washed, stained (GeneChip Fluidics 450 station), and scanned (GeneChip Scanner 3000 G7, GeneChip Command Console v 4.3.3.1616). The transcriptome data analysis was performed with Applied BioSystems Transcriptome Analysis Console software v4.0.2.15 (Genomics Core Facility at Stony Brook University).

### Enzyme-linked immunosorbent assay

To measure endothelial immune responses to rickettsial infections, HMEC-1 or HDMEC were infected with *R. conorii* WT or *pso* variants at an MOI of 3 for 6 hours at 34°C with 5% CO_2_. The supernatant samples were centrifuged (17,000 × *g*, 15 minutes) and double-filtered using 0.22 µm syringe filters (Corning) to remove infectious particles. To determine systemic immune responses to rickettsial infections, mice were infected with 1 × 10^3^ PFU *R. conorii* WT or *pso* variants. After three days of infection, serum samples were collected (Sarstedt), diluted, and double-filtered using 0.22 µm syringe filters (Corning) to remove infectious particles. Sandwich ELISAs were performed to quantify concentrations of CXCL1, CCL2, and IL-6 (Human or Mouse Duoset ELISA kits, R&D systems). Briefly, 96-well microplates were coated with capture antibodies in PBS and incubated overnight at room temperature. The following day, plates were washed with wash buffer (PBS with 0.05% Tween 20) and blocked with blocking buffer (PBS with 1% BSA) for 1 hour. After washing, the plates were incubated with dilutions of standards and experimental samples for 2 hours, detection antibody solutions for 2 hours, and Streptavidin-HRP for 20 minutes in the dark. Plates were incubated at room temperature and washed multiple times between each step. Immune reactive signals were developed (OptEIA, BD Biosciences) and measured at 450 nm, with wavelength correction at 540 nm. Concentrations were calculated with a standard curve using a four-parameter logistic curve fit.

To measure *R. conorii*-specific antibody titers in HK2-infected mice, 96 well plates (Nunc MaxiSorp, Thermo Fisher) were coated with formalin-inactivated *R. conorii* WT (5 × 10^5^ PFU) in 0.1 M carbonate buffer (pH 9.5) and incubated overnight at 4°C. The following day, plates were washed and blocked with PBS containing 5% non-fat milk for 1 hour at room temperature. After washing, plates were incubated with serially diluted hyperimmune mouse sera for an hour at room temperature. Following another washing, plates were incubated with HRP-conjugated anti-mouse IgG (1:10,000 dilution, Rockland or 1:6,000 dilution, Amersham ECL) for an hour at room temperature. Immune reactive signals were developed (OptEIA, BD Biosciences) and measured at 450 nm to calculate half-maximal antibody titers (GraphPad Prism).

### Mouse model of Mediterranean spotted fever

C3H/HeN mice (male and female, 6-week, N = 10 per group, Charles River Laboratories) were infected by intravenous retro-orbital injection with 1 × 10^3^ PFU of *R. conorii* WT or *pso* variants in 0.1 ml SPG. Infected mice were monitored daily for signs of disease and weight loss. Separate cohorts of mice were euthanized at pre-determined time points. Blood was collected via cardiac puncture to collect serum samples. Spleen and lung tissues were harvested and homogenized in SPG with a handheld tissue homogenizer (Omni). Organ homogenates were centrifuged (1,000 × *g*, 5 minutes) to remove cellular debris, and the supernatants were stored at -80°C to determine rickettsial abundance by plaque assay. For the vaccination studies, mice were intravenously immunized with 5 × 10^6^ PFU HK2 in 0.1 ml SPG or mock-immunized with SPG. On day 21 post-immunization, mice were bled and challenged with 1 × 10^3^ *R. conorii* WT. Challenged mice were monitored daily for signs of disease and weight loss.

### Statistical analyses

All statistical analyses were performed using Prism software (GraphPad). Two-way ANOVA with Tukey’s multiple comparisons was performed to determine the statistical significance of rickettsial replication in tissue culture cells, mouse body weight changes, and rickettsial burden in organ homogenates. One-way ANOVA with Tukey’s multiple comparisons was used to analyze the statistical significance of ELISA data. Nonparametric Kruskal-Wallis with Dunn’s multiple comparison test identified the statistical significance of plaque size comparison data. Lastly, Log-rank tests were performed to determine the statistical significance of mouse mortality data.

## Results

### *pso* is required for intracellular invasion and cytopathogenesis in Vero cells

**Fig 1:**
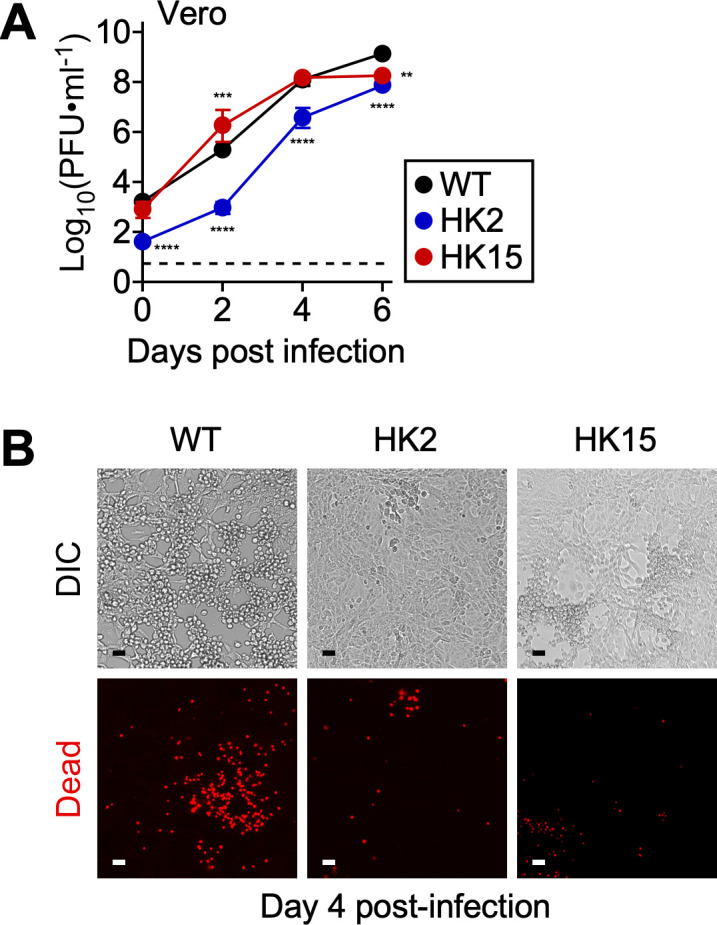
*pso* contributes to rickettsial invasion in Vero cells. (A) Vero cells (N = 3, mean ± standard deviation) were infected with *R. conorii* WT or *pso* variants (HK2 and HK15) at an MOI of 0.01 to determine rickettsial replication at timed intervals. (B) Representative DIC and fluorescent microscopic images of Vero cells infected with *R. conorii* WT or *pso* variants on day 4 post-infection. Two-way ANOVA with Tukey’s multiple comparisons test was performed, ** P < 0.01, *** P < 0.001, **** P < 0.0001.

We utilized two *pso* variants, HK2 and HK15, with *kkaebi* transposon insertions in *Rc0457* (UDP-GlcNAc 4,6-dehydratase/3,5-epimerase) and *Rc0459* (group-specific glycosyltransferase) of *R. conorii* [25]. Previous work documented that *pso* variants synthesize antigenically distinct LPS; HK2 completely lacks O-Ag, while HK15 produces immunologically distinct O-Ag, presumably due to the absence of short side glycan chains. Confirming our earlier findings, HK2 exhibited defective attachment and invasion in Vero cells, resulting in persistently low infectious titers compared to *R. conorii* WT infections (Fig 1A). In contrast, HK15 replicated at a comparable level to *R. conorii* WT. By day 4 pi, *R. conorii* WT caused severe cytopathology and cell death in multiple areas of Vero cells (Fig 1B). In contrast, HK2 and HK15 infections caused minor to moderate levels of cell death and plaque formation. These results validated the importance of *pso* in rickettsial attachment, invasion, and intracellular replication in Vero cells.

### *pso* is required for intracellular invasion and cytopathogenesis in endothelial cells

**Fig 2:**
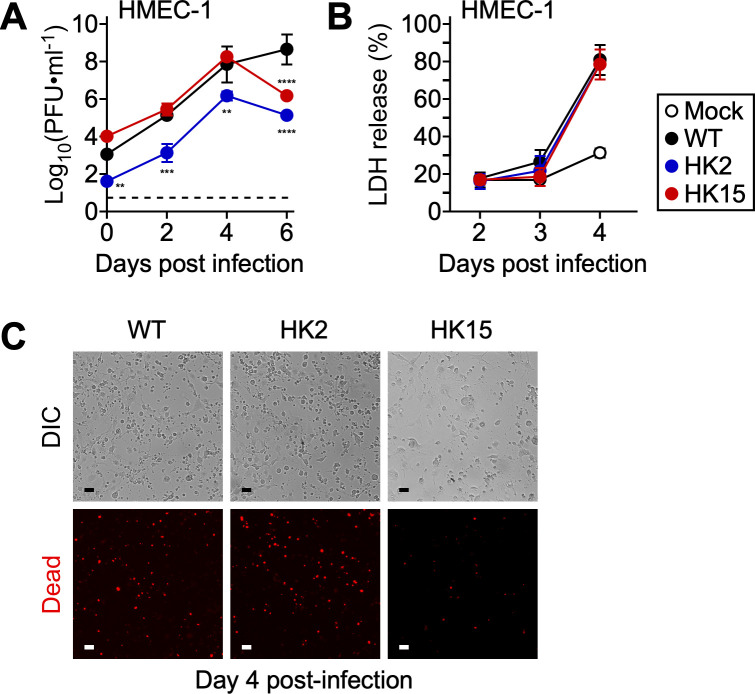
*pso* contributes to rickettsial invasion and intracellular survival in endothelial cells. (A) HMEC-1 cells (N = 3, mean ± standard deviation) were infected with *R. conorii* WT or *pso* variants at an MOI of 0.01 to determine rickettsial replication at timed intervals and (B) LDH release in the supernatant. (C) Representative DIC and fluorescent microscopic images of HMEC-1 cells infected with *R. conorii* WT or *pso* variants on day 4 post-infection. Two-way ANOVA with Tukey’s multiple comparisons test was performed, ** P < 0.01, *** P < 0.001, **** P < 0.0001.

After confirming phenotypes associated with *pso* infections in Vero cells, we utilized HMEC-1 (human microvascular endothelial cells) as a model to assess the contributions of *pso* in endothelial cells. We inoculated HMEC-1 cells with *R. conorii* WT or *pso* variants and determined infectious titers and cytopathology at timed intervals (Fig 2A). *Rickettsia conorii* WT expanded rapidly and reached a maximal titer on day 6 pi. In contrast, HK2 exhibited decreased host adhesion and invasion in HMEC-1 on day 0, leading to significant reductions in plaque numbers in subsequent days. *Rickettsia conorii* WT and HK15 replicated at a comparable rate during the early days of HMEC-1 infections. However, we observed significant reductions in infectious titers for HK2 and HK15 on day 6 [*R. conorii* WT log_10_(PFU ∙ ml^-1^) vs. HK2 log_10_(PFU ∙ ml^-1^), 8.60 ± 0.84 vs. 5.14 ± 0.12, P < 0.0001; *R. conorii* WT log_10_(PFU ∙ ml^-1^) vs. HK15 log_10_(PFU ∙ ml^-1^), 8.60 ± 0.84 vs. 6.19 ± 0.05, P < 0.0001; mean ± standard deviation]. Importantly, in contrast to what we observed in Vero cells, infections with *R. conorii* WT and *pso* variants caused comparable LDH release and cell death in HMEC-1 cells (Fig 2B and 2C), suggesting potential mechanisms by which endothelial cells control intracellular replication of *pso* variants.

### *pso* modulates endothelial cell responses

**Fig 3:**
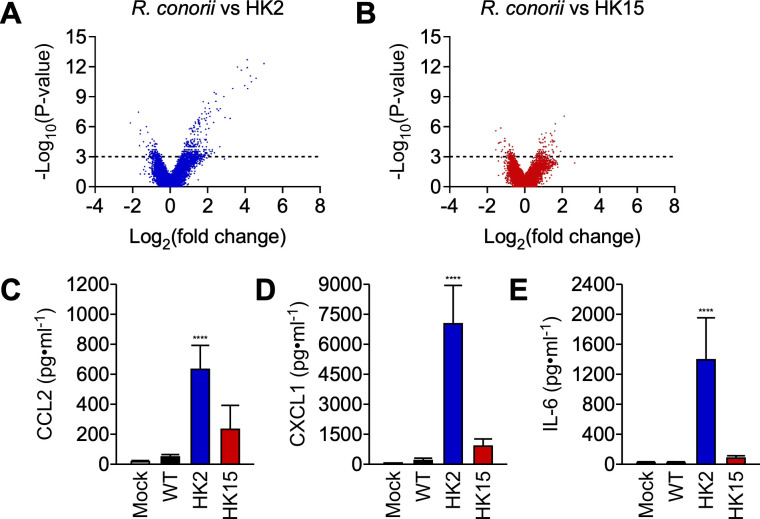
Differential endothelial cell responses to *pso* infections. (A, B) HMEC-1 cells (N = 3, mean ± standard deviation) were infected with *R. conorii* WT or *pso* variants at an MOI of 3 for 6 hours to identify genes differentially regulated upon rickettsial infections. ELISA was performed on the culture supernatant to determine the abundance of (C) CCL2, (D) CXCL1, and (E) IL-6. One-way ANOVA with Tukey’s multiple comparisons test was performed, **** P < 0.0001.

To gain insight into how endothelial cells respond to rickettsial infections, we collected mRNA samples from HMEC-1 cells synchronously infected with *R. conorii* or *pso* (an MOI of 3) at 6 hours pi and performed microarray analysis. This analysis identified 505 (*R. conorii* vs. HK2) and 259 genes (*R. conorii* vs. HK15) differentially regulated in HMEC-1 cells [a fold change greater than 2 or less than -2 (log2 = 1), P < 0.05, Fig 3A and 3B**, and** S1 Table]. Our data suggest that HMEC-1 cells infected with *R. conorii* generated increased levels of transcripts involved in interferon responses, such as IFI44 and IFIT2. On the other hand, *pso* infections induced transcripts responsible for cell adhesion (ICAM1 and VCAM1) and chemokine/cytokine responses (IL-1β, IL-6, CCL2, CXCL1, CXCL8, and CCL20) for immune cell recruitment and local inflammation (Table 1). We performed ELISA to confirm the increased abundance of CCL2, CXCL1, and IL-6 in the supernatant of HMEC-1 cells infected with HK2 but not with *R. conorii* WT or HK15 (Fig 3C, 3D, and 3E). Our data suggest that the HK2 variant lacking O-Ag biosynthesis fails to evade host innate immune mechanisms. Thus, it is intriguing to exploit *pso* variants to identify factors contributing to rickettsial immune evasion mechanisms in endothelial cells.

**Table 1:** Differential gene expressions in *Rickettsia*-infected HMEC-1 cells.

Gene	Name	HK2^1^	HK15^1^	WT^1^
*CXCL1*	Chemokine (C-X-C motif) ligand 1	71.19	5.9	2.96
*ICAM1*	Intercellular adhesion molecule 1	50.79	6.57	2.89
*CXCL8*	Chemokine (C-X-C motif) ligand 8	49.13	4.89	2.83
*CCL2*	Chemokine (C-C motif) ligand 2	41.4	5.54	1.28
*IL1β*	Interleukin 1 beta	41.32	6.12	2.05
*IL6*	Interleukin 6	34.15	2.23	1.97
*CCL20*	Chemokine (C-C motif) ligand 20	28.69	1.38	1.47
*VCAM1*	Vascular cell adhesion molecule 1	13.76	4	2.75
*IFI44*	Interferon-induced protein 44	5.33	9.66	17.18
*IFIT2*	Interferon-induced protein with tetratricopeptide repeats 2	3.6	16.79	15.64

^1^Fold change of gene expression in *Rickettsia*-infected HMEC-1 cells over uninfected control.

### *pso* contributes to rickettsial virulence in primary human endothelial cells

**Fig 4:**
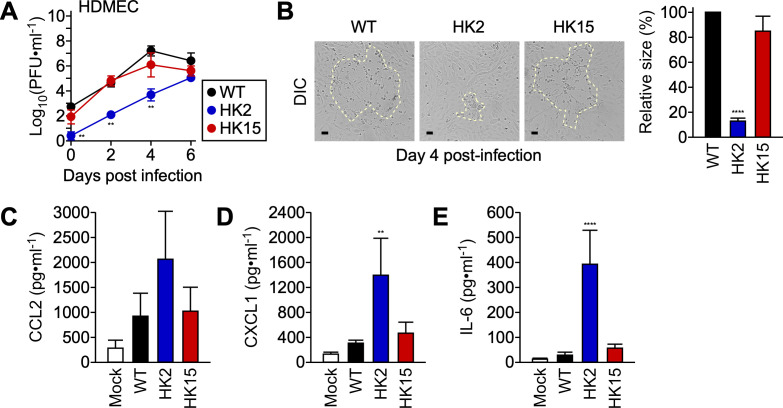
*pso* variants exhibit defective invasion and immune modulation in primary endothelial cells. (A) HDMECs (N = 3, mean ± standard deviation) were infected with *R. conorii* WT or *pso* variants at an MOI of 0.01 to determine rickettsial replication at timed intervals. (B) Representative DIC microscopic images of HDMECs infected with *R. conorii* WT or *pso* variants on day 4 post-infection. Areas exhibiting cytopathology are marked with yellow dotted lines and quantified [N = 25 (WT), 17 (HK2), and 32 (HK15) plaques from 9, 10, and 12 DIC images, mean ± standard error of the mean]. To determine the abundance of (C) CCL2, (D) CXCL1, and (E) IL-6, ELISA was performed on the culture supernatants of HDMECs infected with *R. conorii* WT or *pso* variants at an MOI of 3 for 6 hours. One-way or Two-way ANOVA with Tukey’s multiple comparisons test or nonparametric Kruskal-Wallis with Dunn’s multiple comparison test were performed, ** P < 0.01, **** P < 0.0001.

Earlier studies documented that HMEC-1 cells exhibit key attributes of endothelial cells, serving as a model for studying *Rickettsia*-endothelial cell interactions [31,32]. Since HMEC-1 cells are immortalized microvascular endothelial cells, we characterized *R. conorii pso* infections in primary human dermal microvascular endothelial cells (HDMEC). Upon infection, *R. conorii* WT expanded rapidly, generating severe cytopathology and plaque formation starting on day 4 of infection (Fig 4A and 4B). By day 6 of infection, all HDMECs succumbed to *R. conorii* infection and became non-adherent. Similarly, HK15 replicated at a comparable rate, damaging HDMECs with distinct plaques on day 4 pi. In contrast, HK2 exhibited defective abilities to invade and replicate in HDMECs with significantly lower infectious titers and minimal cytopathology. Importantly, we observed similar patterns of chemokine and cytokine inductions from HK2-infected HDMECs, confirming that the HK2 variant fails to dampen endothelial cell responses (Fig 4C, 4D, and 4E). Our data suggest that HK2-infected endothelial cells mount robust inflammatory responses and secrete elevated levels of chemoattractant, potentially leading to rapid recruitment and activation of phagocytes, such as macrophages and neutrophils.

### *pso* is required for intracellular survival in macrophages

**Fig 5:**
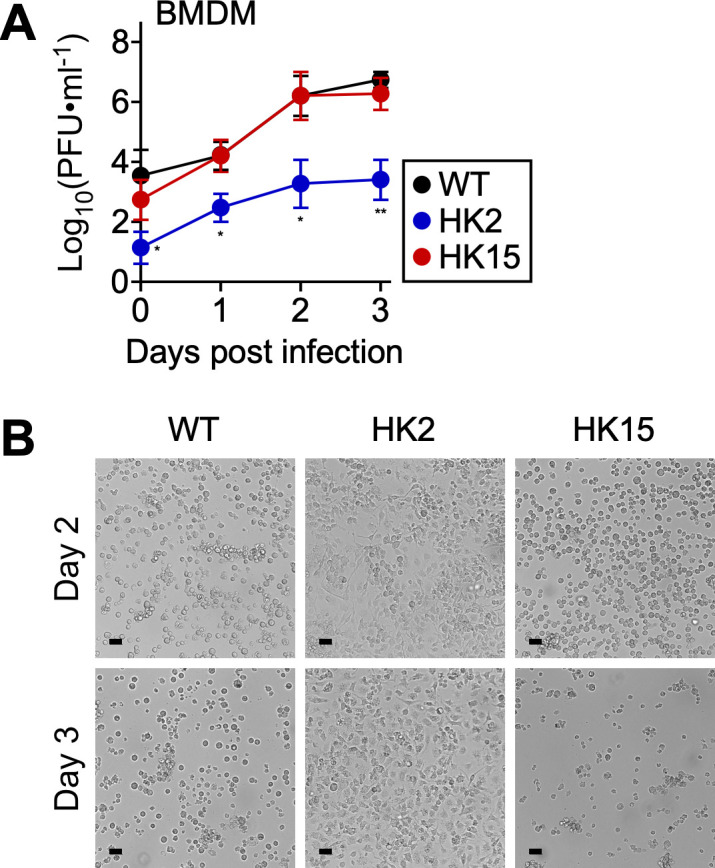
*pso* variant HK2 is defective for attachment and survival in BMDMs. (A) BMDMs (N = 3, mean ± standard deviation) were infected with *R. conorii* WT or *pso* variants at an MOI of 0.1 to determine rickettsial replication at timed intervals. (B) Representative DIC microscopic images of BMDMs infected with *R. conorii* WT or *pso* variants on days 2 and 3 post-infection. Two-way ANOVA with Tukey’s multiple comparisons test was performed, * P < 0.05, ** P < 0.01.

Recent studies have documented that pathogenic rickettsial species evade bactericidal mechanisms and survive within macrophages. Thus, we performed a comparative growth analysis of *R. conorii* WT and *pso* variants in BMDMs (Fig 5A and 5B). Upon infecting BMDMs at an MOI of 0.1, *R. conorii* WT and HK15 expanded rapidly and caused severe cytopathology on days 2 and 3 pi. In contrast, HK2 struggled to invade and replicate in BMDMs with minimal cytopathology over three days of infection [*R. conorii* WT log_10_(PFU ∙ ml^-1^) vs. HK2 log_10_(PFU ∙ ml^-1^), 6.22 ± 0.63 vs. 3.25 ± 0.79, P < 0.05 on day 2; *R. conorii* WT log_10_(PFU ∙ ml^-1^) vs. HK2 log_10_(PFU ∙ ml^-1^), 6.71 ± 0.29 vs. 3.43 ± 0.62, P < 0.01 on day 3; mean ± standard deviation]. Our results confirm prior findings that pathogenic *Rickettsia* species dampen host innate immune responses for successful intracellular survival in BMDMs and suggest that macrophages recruited to the HK2-infected endothelial cells restrict rickettsial replication and spread.

### *pso* variants exhibit virulence defects in the mouse model of spotted fever

**Fig 6:**
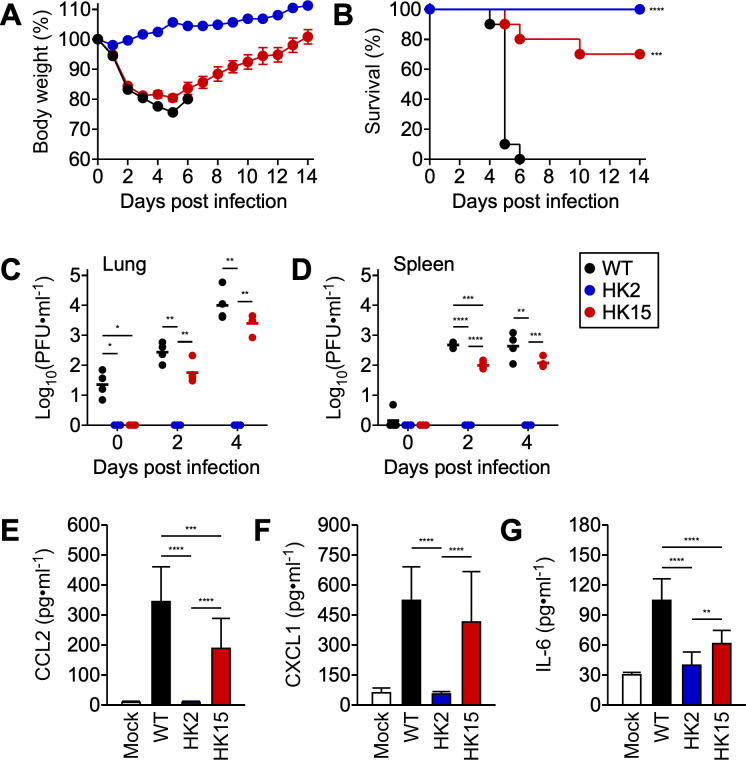
*pso* contributes to rickettsial pathogenesis in mice. Cohorts of C3H mice (N = 10) were intravenously injected with 1 × 10^3^ PFU *R. conorii* WT or *pso* variants and monitored for 14 days to document (A) body weight changes (mean ± standard error of the mean) and (B) mortality. On days 0, 2, and 4 of infection, rickettsial burdens in the (C) lungs and (D) spleen were assessed by plaque assay (N = 4). To determine the abundance of (E) CCL2, (F) CXCL1, and (G) IL-6, ELISA was performed with hyperimmune sera collected from *Rickettsia*-infected mice (N = 10) on day 3 of infection. One-way or Two-way ANOVA with Tukey’s multiple comparisons and Log-rank test were performed. * P < 0.05, ** P < 0.01, *** P < 0.001, **** P < 0.0001.

Our experimental results suggest that *R. conorii* can subvert innate immune mechanisms during the initial phase of intracellular replication in endothelial cells. In contrast, the HK2 variant fails to control endothelial cell responses, leading to the secretion of inflammatory mediators. This, in turn, will alert other professional immune cells and result in rapid clearance of HK2. To test this hypothesis, cohorts of C3H mice were infected with 1 × 10^3^ PFU *R. conorii* WT or *pso* variants (Fig 6A and 6B). All mice infected with *R. conorii* WT displayed signs of acute disease, amounting to a significant drop in body weight by day 5 pi, with lethal outcomes. In contrast, all HK2-infected mice survived with minimal signs of clinical symptoms (*R. conorii* WT vs. HK2, P < 0.0001). HK15 infections caused acute body weight loss with 30% mortality over 14 days of infection (*R. conorii* WT vs. HK15, P < 0.001). We determined infectious titers in the lungs and spleen with plaque assays during the acute phase of infections (Fig 6C and 6D). In the lungs of mice infected with *R. conorii* WT, rickettsial burdens rapidly increased from 1.36 ± 0.43 [log_10_(PFU ∙ ml^-1^), mean ± standard deviation] on day 0 pi to 4.01 ± 0.53 [log_10_(PFU ∙ ml^-1^), mean ± standard deviation] on day 4 pi, with persistent rickettsial survival, reaching to 2.63 ± 0.46 [log_10_(PFU ∙ ml^-1^), mean ± standard deviation] in the spleens on day 4 pi. Similarly, HK15 infections increased rickettsial burdens in the lungs [3.38 ± 0.31, log_10_(PFU ∙ ml^-1^), mean ± standard deviation] and spleen [2.07 ± 0.16, log_10_(PFU ∙ ml^-1^), mean ± standard deviation] on day 4 pi. However, rickettsial burdens in HK15-infected mice were significantly lower than those of *R. conorii* WT-infected mice. Most strikingly, all organ homogenates of HK2-infected mice did not produce infectious titers, suggesting that HK2 failed to seed productive infectious foci in mice. We performed ELISA to determine systemic immune responses to rickettsial infections and quantified the abundance of CCL2, CXCL1, and IL-6 in immune sera during the acute phase (Fig 6E, 6F, and 6G). In contrast to *in vitro* observations with endothelial cells, we detected significantly increased levels of CCL2, CXCL1, and IL-6 in mice infected with *R. conorii* WT and HK15 (day 3 pi), positively correlating with their virulence in mice. Of note, HK2 infections elicited *R. conorii*-specific antibody responses, with half-maximal titers of 590 ± 48 (mean ± standard error of the mean, day 14 pi), suggesting successful, yet transient, HK2 infections for immune recognition in mice. Our results confirm that LPS biosynthesis is directly linked to rickettsial immune evasion and virulence.

### HK2 elicits protective immunity against the *R. conorii* challenge

**Fig 7:**
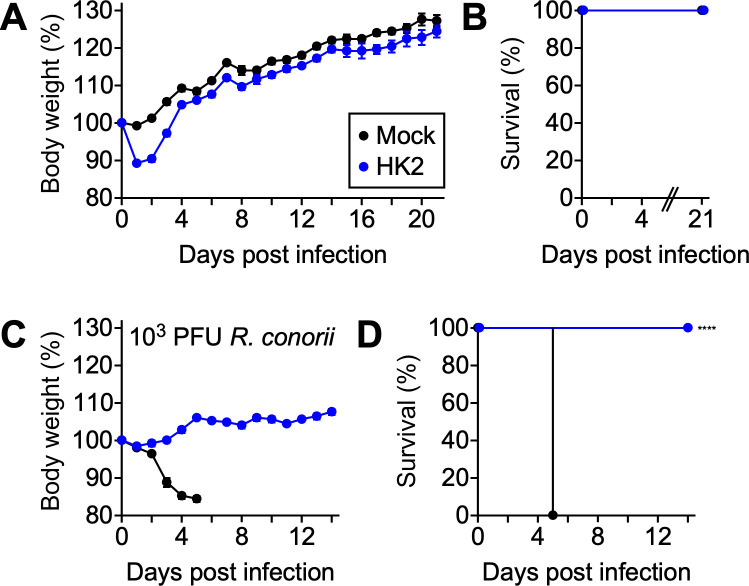
*pso* variant HK2 is avirulent and functions as a live-attenuated vaccine. Cohorts of C3H mice (N = 5) were intravenously injected with 5 × 10^6^ PFU HK2 or SPG buffer (Mock) and monitored for 21 days to document (A) body weight changes (mean ± standard error of the mean) and (B) mortality. On day 21 post-infection, surviving mice were challenged with 1 × 10^3^ PFU *R. conorii* WT to determine (C) body weight changes (mean ± standard error of the mean) and (D) mortality. Log-rank test was performed. **** P < 0.0001.

Inactivated whole-cell and live-attenuated vaccine strategies have been evaluated to provide immune protection against fatal rickettsioses [33–37]. Unfortunately, these vaccines have been shown to generate insufficient immunogenicity, limited protective efficacy, or potential adverse outcomes from revertant infections in human volunteers [37–39]. Despite the technical challenges, these studies established the groundwork for understanding the immunopathological consequences of rickettsial infections and vaccine-mediated immune protection. Here, we utilized the HK2 variant as a live-attenuated vaccine to unveil the biological roles of *pso* in eliciting immune protection against subsequent infections in the mouse infection model. As expected, the HK2 variant displayed severe virulence defects, allowing mice infected with a high dose (5 × 10^6^ PFU HK2) to survive for 21 days (Fig 7A and 7B). Animals exhibited mild body weight loss (<12%) within 2 days, followed by a full recovery by day 4 pi. All HK2-immunized mice have successfully seroconverted with *R. conorii*-specific half-maximal titers of 1,262 ± 168 (mean ± standard error of the mean, day 21 pi). When challenged with a lethal dose of *R. conorii* WT, HK2-immunized mice did not lose significant body weight and failed to develop clinical symptoms (Fig 7C and 7D). In contrast, mock-immunized animals succumbed to the infection by day 5 pi. These results suggest that *pso* variants provide a unique opportunity to investigate the cellular and humoral immune components associated with protective immunity and immune evasion mechanisms associated with rickettsial pathogenesis.

## Discussion

*Rickettsia* exhibits a strong tropism for vascular endothelial cells, leading to progressive endothelial dysfunction and vascular damage [40,41]. Thus, understanding how pathogenic *Rickettsia* modulates endothelial cells has significant potential to identify molecular interactions that could be exploited to develop novel therapeutics or vaccines to prevent fatal rickettsial infections. In this study, we sought to investigate how *pso* contributes to rickettsial intracellular survival and influences endothelial immune responses to rickettsial infections. By studying microvascular endothelial cells infected with *R. conorii pso* variants, we determined that the inability to synthesize wild-type O-Ag is directly associated with defective virulence and immune modulation of endothelial cells.

Endothelial cells are sentinel gatekeepers with multiple immune regulatory functions to detect pathogen/damage-associated molecular patterns, modulate local and systemic inflammation, recruit professional immune cells, and orchestrate host immunity against bloodborne microbial pathogens [10,42]. On the other hand, being an obligate intracellular pathogen, *Rickettsia* must have evolved to shield against, actively evade, or take advantage of innate defense mechanisms designed to detect pathogen-derived molecular structures, such as peptidoglycan fragments and LPS, released during intracellular replication. Previous studies have documented this complex tug-of-war occurring between *Rickettsia* and endothelial cells. For instance, endothelial cells infected with *R. rickettsii* produce IL-1α, while those invaded by *R. conorii* or *R. rickettsii* release IL-8 and CCL2 [43–47]. In addition, endothelial cells exposed to *R. conorii* secrete IL-6 in an IL-1α-dependent manner and increase the expression of adhesion molecules, such as ICAM-1 and VCAM-1 [43,47,48]. It has been reported that endothelial cells can recognize extracellular LPS through the TLR4-MD2-CD14 receptor complex and activate downstream proinflammatory signaling pathways [49–51]. Further, endothelial cells utilize caspase-4/5 (human) or caspase-11 (mouse) to detect intracellular LPS, activating NLRP3 inflammasome and GSDMD-mediated pyroptotic cell death [52,53]. Although the endotoxic potential of rickettsial LPS remains unclear, previous research documented that rickettsial infections can activate TLR4/MD-2 signaling and noncanonical inflammasome pathways. Thus, understanding the mechanistic details of how rickettsial LPS activates extracellular and intracellular immune signaling pathways in endothelial cells has significant potential to uncover their biological impacts on rickettsial pathogenesis.

Our microarray analysis corroborates earlier findings that *R. conorii* infections induced differential gene expression in endothelial cells. Importantly, we observed distinct alterations in *pso*-infected endothelial cells. Infections with *pso* variants (especially with the HK2 variant lacking O-Ag biosynthesis) activated endothelial cells with abundant expression of cell adhesion molecules (ICAM-1 and VCAM-1) and inflammatory mediators (CCL2, CXCL1, CXCL8, CCL20, and IL-6). The chemokines (CCL2, CXCL1, CXCL8, and CCL20) facilitate the recruitment of monocytes and neutrophils to the site of infection [54–57]. Interaction with two cell adhesion molecules, ICAM-1 and VCAM-1, aids in the transmigration and activation of immune cells [58–60]. Proinflammatory cytokines, such as IL-6 and IL-1β, activate recruited immune cells, initiate systemic responses, and induce bactericidal responses [61,62]. In contrast, endothelial cells harboring intracellular *R. conorii* WT expressed increased levels of two interferon-stimulated genes (ISG), IFI44 and IFIT2. Recent studies documented that IFI44 induces anti- or pro-viral activities depending on the invading microorganisms [63,64]. Similarly, IFIT2, one of the IFN-induced proteins with tetratricopeptide repeats (also known as ISG54/P54), recognizes AU-rich regions in cellular and viral RNAs to regulate and drive anti- or pro-viral functions depending on the viral pathogens [65,66]. In addition, IFIT2 has been associated with the amplification of proinflammatory IFN signaling cascades initiated by LPS and the induction of BCL-2-driven intrinsic apoptosis [67,68]. Prior studies suggested *Rickettsia* battles against host cells to regulate cell death pathways, potentially compromising host defense mechanisms against rickettsioses [69,70]. To our surprise, despite significant differences in virulence, infections with *R. conorii* WT and *pso* variants caused similar levels of cell death in HMEC-1 cells. Our data suggest that *R. conorii* requires *pso* to actively suppress endothelial cell-mediated inflammation and delay cell death pathways to protect its replicative niche within endothelial cells. Thus, understanding how *pso*-driven immune activation controls rickettsial intracellular replication may provide insights into identifying the underlying molecular interactions between *Rickettsia* and endothelial cells.

Recent studies have shown that disease severity correlates with *Rickettsia’s* ability to survive and proliferate in macrophages. Pathogenic species, including *R. rickettsii*, *R. conorii*, *Rickettsia parkeri*, and *Rickettsia helvetica*, have evolved sophisticated strategies to manipulate host defenses and persist within macrophages, whereas nonpathogenic species, such as *Rickettsia montanensis*, fail to escape the phagolysosome [28,71,72]. Our experiments with BMDMs corroborated these findings. *Rickettsia conorii* and the HK15 mutant expanded rapidly and caused severe cytopathology, while HK2 struggled to survive with minimal cytopathology. Thus, our results provide a platform to unveil the molecular mechanisms that pathogenic rickettsial species exploit to survive within hostile phagocytes. Further, additional experiments are necessary to determine how activated endothelial cells modulate macrophage-mediated rickettsial clearance.

Infection with *R. conorii* WT caused lethal outcomes with substantial body weight loss, whereas all HK2-infected mice survived with minimal clinical symptoms. Notably, HK2-infected mice showed no infectious rickettsial titers in organ homogenates after 4 days of infection. Conversely, despite significant reductions of infectious titers in organ burdens, HK15 infections led to acute and lethal diseases in mice. By assessing the presence of inflammatory signaling molecules in the serum, we found a positive correlation between rickettsial virulence and inflammatory markers in mice. These observations contrast with our cytokine measurements of *in vitro* endothelial cell infections. Of note, despite lower intracellular bacterial abundance, HK2-infected endothelial cells produced significantly higher levels of inflammatory signaling molecules than those infected with *R. conorii* WT. Thus, we hypothesize that the *in vivo* mouse infection with HK2 may have caused transient immune activation in a limited number of endothelial cells, posing technical difficulties in capturing such changes in the serum. Corroborating our hypothesis, all HK2-infected mice successfully seroconverted with antibodies recognizing *R. conorii* surface molecules.

Our *in vivo* and *in vitro* analyses suggest that, during the initial stages of infection in endothelial cells, *R. conorii* effectively subverts innate immune mechanisms while establishing a replicative niche. As rickettsial replication and dissemination continue, endothelial dysfunction activates robust host defense mechanisms, exacerbating rickettsial pathogenesis. However, in the case of HK2, the inability to synthesize O-Ag compromised the surface protein assembly, inadvertently affecting the rickettsial ability to shield against innate immune surveillance and defense mechanisms. As a result, during the early stages of HK2 infection, endothelial cells respond effectively and orchestrate the recruitment and activation of immune cells, such as macrophages and neutrophils, thereby restricting rickettsial replication and spread. Of note, our previous work documented that the HK2 variant displays increased levels of OmpA/B passenger and β-barrel domains on the outer membrane compared to *R. conorii* WT and HK15 [25]. Additionally, the HK2 β-barrel domain exhibited faster mobility, suggesting that the O-Ag synthesis defect may impact outer membrane protein post-translational modification, surface protein assembly, or enzymatic processing of surface cell antigens, many of which directly contribute to rickettsial pathogenesis and immune evasion. In addition, *Rickettsia* may utilize *pso* for the biosynthesis of the slime layer or uncharacterized glycans. Thus, additional studies are required to determine the direct and indirect biological attributes of *pso* in modulating rickettsial biology and host immune responses during rickettsial intracellular replication. Nevertheless, our experimental data suggest that *pso* is indispensable for rickettsial survival and immune evasion in endothelial cells.

Intravenous infections with HK2 elicited robust immune protection against *R. conorii* in mice. However, it remains to be established if the live-attenuated vaccination induces protective immunity against tick-transmitted *R. conorii* infection. Furthermore, additional studies are needed to determine the minimum effective dose and the immunological basis for vaccine-induced protection. The present study also paves the way forward in initiating studies to characterize additional *pso* mutants expressing chemically distinct LPS molecules, understand the role of LPS in triggering endothelial cell responses, identify host factors involved in detecting *pso* infections, and uncover protective antigens for developing a vaccine for *Rickettsia*.

## Supporting information

S1 Table Differential gene expressions in *Rickettsia*-infected HMEC-1 cells. Fold change of genes differentially expressed in *Rickettsia*-infected HMEC-1 cells over uninfected controls or between infected cells.
(XLSX)

S1 Fig Schematic diagram of endothelial cell responses to rickettsial infections. *Rickettsia conorii* requires O-antigen polysaccharides to modulate endothelial cell responses during intracellular replication. Image Credit: Smruti Mishra and Hwan Keun Kim The referenced image can be published under the Creative Commons Attribution License. The image is created by Biorender (www.biorender.com).
(JPEG)
