# Supplementary figures and images for: Polysaccharide synthesis operon modulates Rickettsia-endothelial cell interactions
Source: PLoS Pathog. 2025 Jun 26;21(6):e1013277. doi: 10.1371/journal.ppat.1013277 (PMC12201665; doi:10.1371/journal.ppat.1013277)

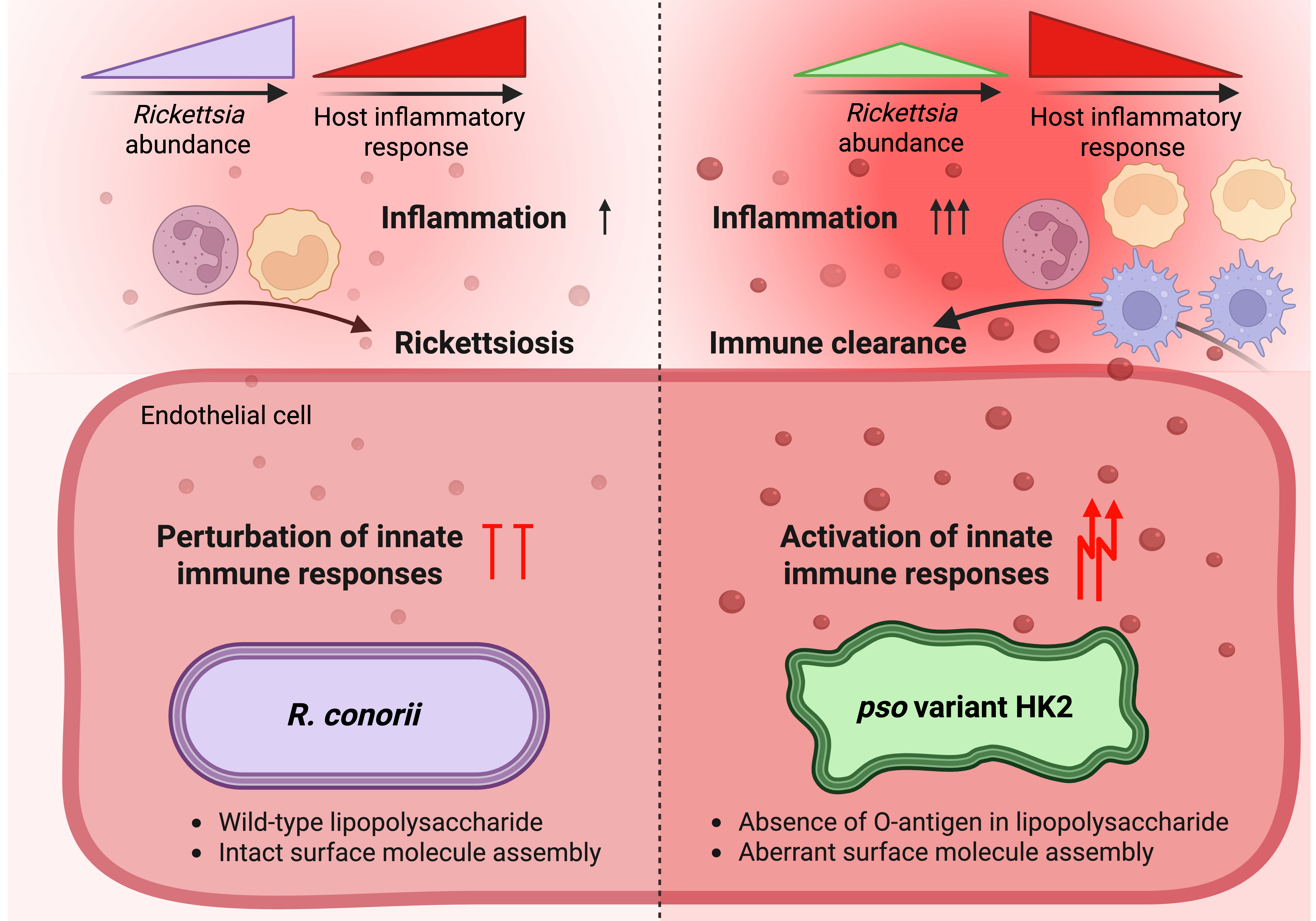

Supplement: S1 Fig — Rickettsia conorii requires O-antigen polysaccharides to modulate endothelial cell responses during intracellular replication. Image Credit: Smruti Mishra and Hwan Keun Kim The referenced image can be published under the Creative Commons Attribution License. The image is created by Biorender (www.biorender.com). (JPEG) [file ppat.1013277.s002.jpeg]
